# Using platelet concentrates to treat maxillofacial tissue lesions

**DOI:** 10.3389/fbioe.2024.1523225

**Published:** 2025-01-07

**Authors:** Ruijia Liu, Siqi Chen, Peng Wang, Ruiye Bi

**Affiliations:** ^1^ State Key Laboratory of Oral Diseases, Sichuan University, Chengdu, China; ^2^ National Clinical Research Center for Oral Diseases, Department of Orthognathic and TMJ Surgery, West China Hospital of Stomatology, Sichuan University, Chengdu, China

**Keywords:** platelet concentrates, platelet-rich plasma, platelet-rich fibrin, bone regeneration, soft tissue regeneration

## Abstract

**Objectives:**

Platelet concentrates (PCs), which are blood products that are abundant in platelets and growth factors, have become pivotal in treating maxillofacial tissue lesions due to their capacity for promoting bone and soft tissue recovery. This review will provide some recent progress of the use of platelet concentrates to treat lesions on maxillofacial tissues.

**Subjects:**

We reviewed the mechanisms by which PCs promote wound healing and tissue recovery and summarized the application of PCs in the treatment of lesions on maxillofacial tissues, including medication-related osteonecrosis of the jaw, post-extraction wound healing, implant surgery, temporomandibular joint diseases, and periodontal tissue restoration.

**Results:**

PC promotes the attachment and proliferation of osteoblasts, as well as the synthesis and deposition of collagen fibers by stimulating the AFK pathway and releasing growth factors and cytokines, such as secreting GFs, VEGF, TGF-β, etc. They also induce angiogenesis, inhibit bone resorption, promote the healing of soft tissues, relieve symptoms, reduce postoperative complications and maintain implant stability.

**Conclusion:**

PCs may be used as an adjuvant therapy in the treatment of lesions on maxillofacial tissues. However, more studies should refine the preparation and treatment methods for platelet concentrates and establish a foundation for their extensive application.

## Introduction

**FIGURE 1:**
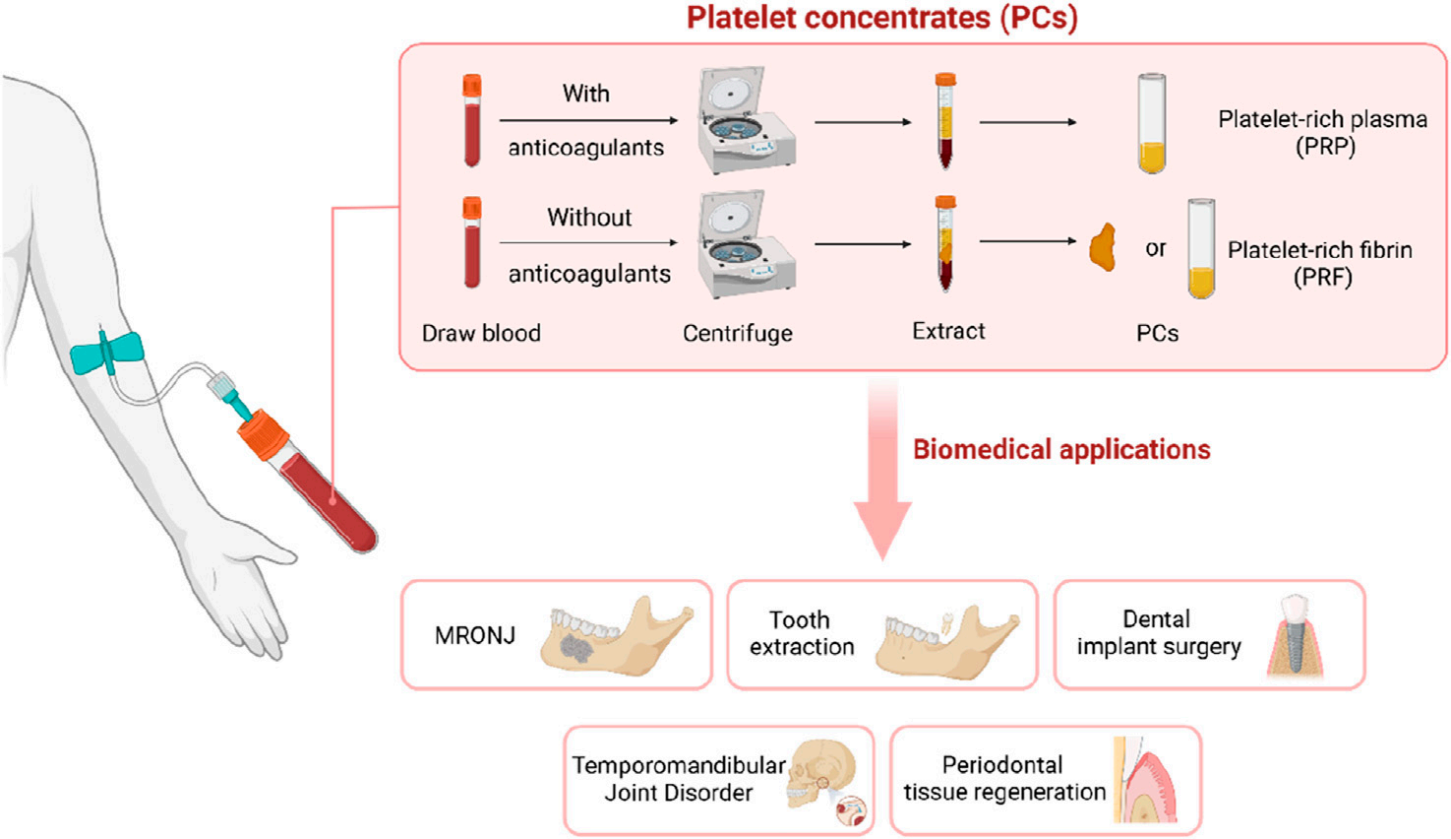
Platelet concentrates (PCs) are extracted by centrifugation of blood. It can be used in MRONJ, tooth extraction, dental implant surgery, temporomandibular joint disorder and periodontal tissue regeneration.

Platelet concentrates (PCs), which are rich in platelets and growth factors (GFs), have played a significant role in the treatment of maxillofacial tissue lesions in recent years. PCs represent refined blood products that are teeming with platelets and an array of bioactive growth factors. Platelet concentrates can be categorized based on their fibrin content, yielding platelet-rich plasma (PRP) and platelet-rich fibrin (PRF). PRP, as a first-generation platelet concentrate, involves centrifugation-enriched autologous blood with added anticoagulants to extract a three-to-fourfold increase in platelet count beyond that found in regular human plasma. Following the activation of platelets and the degranulation of alpha granules, the effusion of growth factors and cytokines ensues, thereby fostering an inflammatory response, bone remodeling, stem cell differentiation, extracellular matrix formation, and the upregulation of related genes (Gaissmaier et al., 2008). Given the limitations associated with anticoagulant constituents in PRP, Choukrun’s subsequent research in the early 2000s focused on developing a second-generation platelet concentrate devoid of anticoagulants (Dohan et al., 2006). This resulted in PRF, which maintains similar attributes to PRP, but can more effectively promote osteogenesis. PRF can be centrifuged directly without adding an anticoagulant after sample collection, and can be prepared in a membranous or semi-fluidic state (Vasilikos et al., 2021). In contrast to PRP, PRF's appeal lies in its expedited, straightforward, and cost-effective production, rendering it an enticing product (Figure 1).

The inherent constituents of PCs can significantly impact their therapeutic efficacy. Their leukocytic concentration delineates them into the pure forms (P-PRP and P-PRF) or the leukocyte-rich variants (L-PRP and L-PRF). L-PRP can significantly promote immune regulation and tissue repair and regeneration, and it can be used to repair bone and soft tissue injuries, especially in cases of infection. However, some scholars argue that specific leukocytes release pro- and anti-inflammatory molecules, playing a crucial role in the inflammatory process preceding tissue regeneration, which may exacerbate swelling and pain (Lana et al., 2019). Therefore, they recommend prioritizing the use of Leukocyte-Poor platelet-rich plasma in the treatment of osteoarthritis (OA) (Filardo et al., 2012). Additionally, aging can also impact the therapeutic efficacy of platelet concentrates. In addition to the constituents of PCs, the process of individual aging also affects their therapeutic effects. Aging leads to reduced cellular stemness and the depletion of growth factor receptor abundance. An animal study showed that P-PRG derived from young horses presented significantly higher PDGF-BB concentrations (P < 0.001) compared to P-PRG from older horses (Giraldo et al., 2013). Compared with their elderly counterparts, the youthful cohort’s larger assemblage of growth factor receptors and augmentation in growth factors elicited a more potent cellular reaction in response to PRP intervention (Wang, 2016). This observation provides an explanation for the underlying rationale behind the comparatively less-effective, and possibly ineffective, outcomes of PRP treatment among the elderly population, who possess fewer and suboptimal reserves of stem cells (Wang, 2014). The growth factors and cytokines including, but not limited to, vascular endothelial growth factor, transforming growth factor-b1, platelet-derived growth factor, epidermal growth factor, hepatocyte growth factor, fibroblast growth factor, and insulin-like growth factor (Eskan et al., 2014), stored in platelets promote the recovery of bone and soft tissues, offering significant benefits in the treatment of maxillofacial tissue lesions. This review aims to summarize the impact of platelet concentrates in the treatment of tissue lesions in different oral and maxillofacial regions.

## Medication-related osteonecrosis of the jaw

Medication-related osteonecrosis of the jaw (MRONJ) is a potential complication observed in patients receiving medications such as bisphosphonates or monoclonal antibodies, which are used for antiresorptive and anti-angiogenic therapies. The most important factor in its pathogenesis is the inhibition of osteoclast activity and bone remodeling induced by anti-bone absorption or anti-angiogenic drugs (Ruggiero et al., 2009).

### Wound healed after MRONJ

**FIGURE 2:**
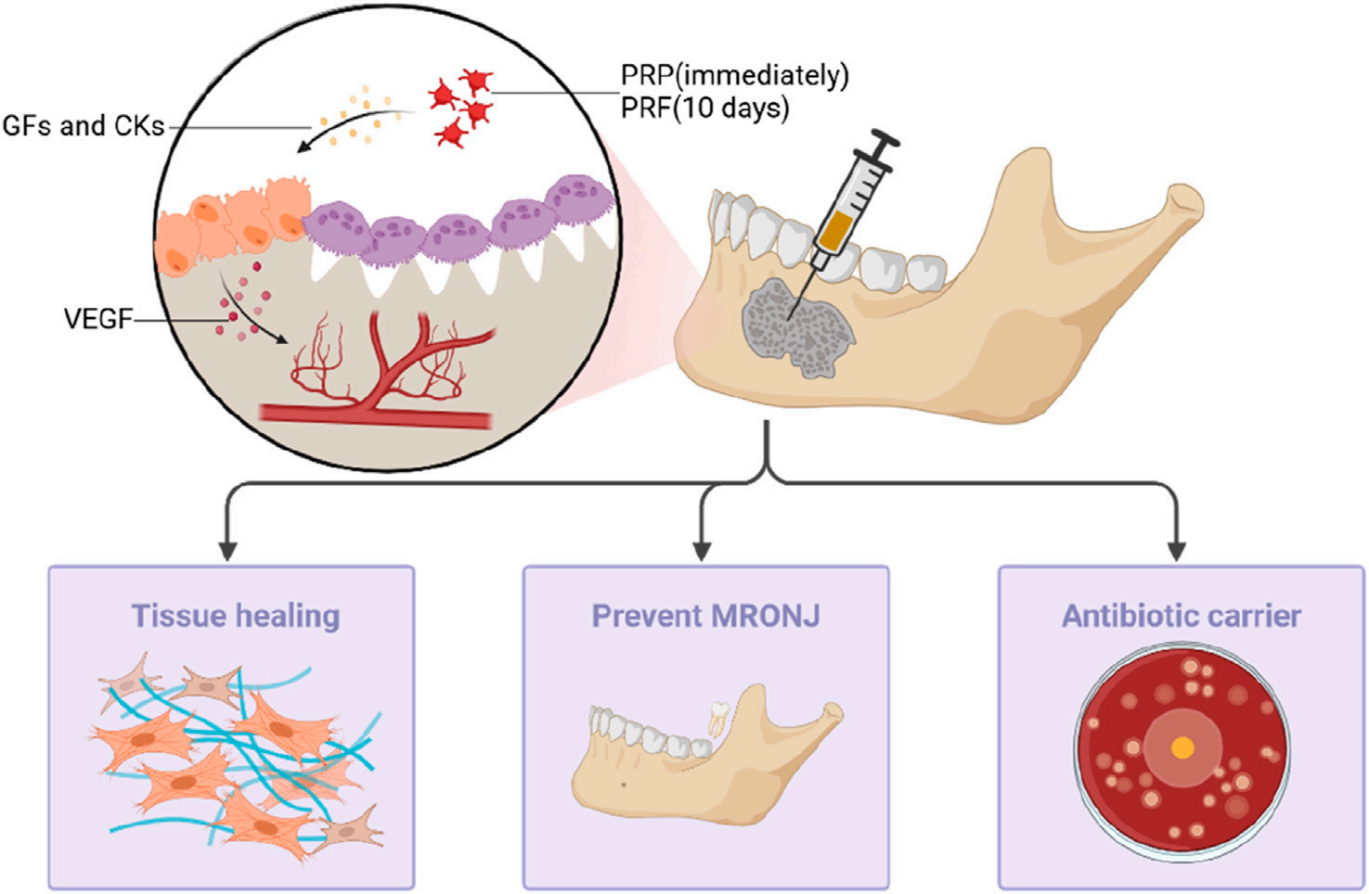
PRP and PRF can prevent MRONJ after tooth extraction by inducing the release of growth factors and cytokines, promoting angiogenesis and healing of bone and mucosal tissues, and can also be used as antibacterial antibiotic biological carriers in the treatment of patients.

Many authors have advocated for the utilization of PRP to augment post-surgical wound healing, particularly in the context of MRONJ surgical interventions. PRP induces the release of growth factors, facilitates angiogenesis, and fosters the healing of both bone and mucosal tissues, thereby accelerating the recovery of patients undergoing bisphosphonate treatment (Marcazzan et al., 2018) (Figure 2). In Cardoso et al. (2019)’s study on rats undergoing tooth extraction during bisphosphonate treatment, they demonstrated that the group treated with PRP showed more new bone formation, increased vascularization, and a slightly higher expression level of VEGF in histological analysis. This provide compelling evidence of the beneficial impact of PRP on the resolution of the bisphosphonate-related osteonecrosis of the jaw (BRONJ). In clinical practice, PRP has demonstrated efficacy in treating and preventing the recurrence of osteonecrosis of the jaw. Mauceri et al. (2018) conducted a study involving ten patients with BRONJ who underwent a conservative surgical treatment combining an Er,Cr:YSGG laser and PRP for the treatment. The authors observed that following the combined treatment, the clinical and imaging signs of BRONJ recurrence were absent in 30% of the patients, while 50% exhibited clinical improvement.

PRF can release numerous cytokines and growth factors that can facilitate osteoblast differentiation and promote bone maturation (Goto et al., 2006). Compared to PRP, L-PRF can act for longer (He et al., 2009). Some *in vitro* studies indicate that PRF gradually releases growth factors (GFs) and cytokines over at least 10 days, while PRP gel exhibits different kinetics, releasing most of the GFs and bioactive molecules immediately after activation and within 6–8 h after application (Kobayashi et al., 2016). Due to its high expression of leukocytes within the fibrin matrix, PRF can overcome infection at sites where the healing process is challenging. The effectiveness of PRF as a standalone treatment is debated. Blatt et al. (2022) quantitatively analyzed their samples and found that it may not significantly optimize wound healing; no significant changes were found in the subjects’ health status, pain sensation, and oral health-related quality of life. However, combining PRF with various therapeutic modalities, such as surgery, L-PRF, and photobiomodulation (PBM), can lead to notable improvements in the treatment outcomes. The main reasons for this success are that systemic antibiotics can control microbial growth, L-PRF plays a positive role in tissue healing, and the biophysical properties of PBM induce cell proliferation and enhance stem cell differentiation (Tenore et al., 2020).

### Prevention of MRONJ after tooth extraction

In addition to assisting in the treatment of MRONJ, PRF can also prevent MRONJ after tooth extraction. Tooth extraction surgery has been identified as the primary risk factor for MRONJ, particularly among patients with systemic diseases. MRONJ prophylaxis after tooth extraction can be facilitated through the application of PCs. In 2021, Razmara et al. (2021) conducted experiments on male Wistar rats, revealing that the application of collagen scaffolds enriched with PRP is an efficacious prophylactic measure for MRONJ. This approach influences factors such as the quantity of osteoblasts and osteoclasts, bone density, new bone formation, inflammation, and osteonecrosis. In clinical trials, the use of PRF during tooth extraction procedures on patients undergoing treatment with antiresorptive or anti-angiogenic drugs can effectively prevent MRONJ. Miranda et al. (2021) found that among patients receiving antiresorptive or anti-angiogenic drugs, none of the individuals in the group with a PRF plug inserted into the extraction socket had MRONJ, whereas the group without the PRF insertion had a 19.23% incidence of MRONJ. Poxleitner et al. (2020)’s study indicated that in patients with osteoporosis undergoing tooth extraction while receiving antiresorptive therapy, the use of PRF without subsequent primary closure demonstrated comparable efficacy in preventing osteonecrosis of the jaw to that of primary closure with a mucoperiosteal flap.

### Antibacterial antibiotic biological carrier

PRF is a potent antibiotic biological carrier, exhibiting robust antibacterial capabilities. In a study involving 24 patients with osteonecrosis of the jaw undergoing systemic antibiotic therapy with ampicillin/sulbactam, Straub et al. (2022) ascertained PRF’s potential as an antibiotic biological carrier. However, further research is needed to explore the duration of its effects and the kinetics of its release.

## Tooth extraction

### Alveolar ridge preservation

**FIGURE 3:**
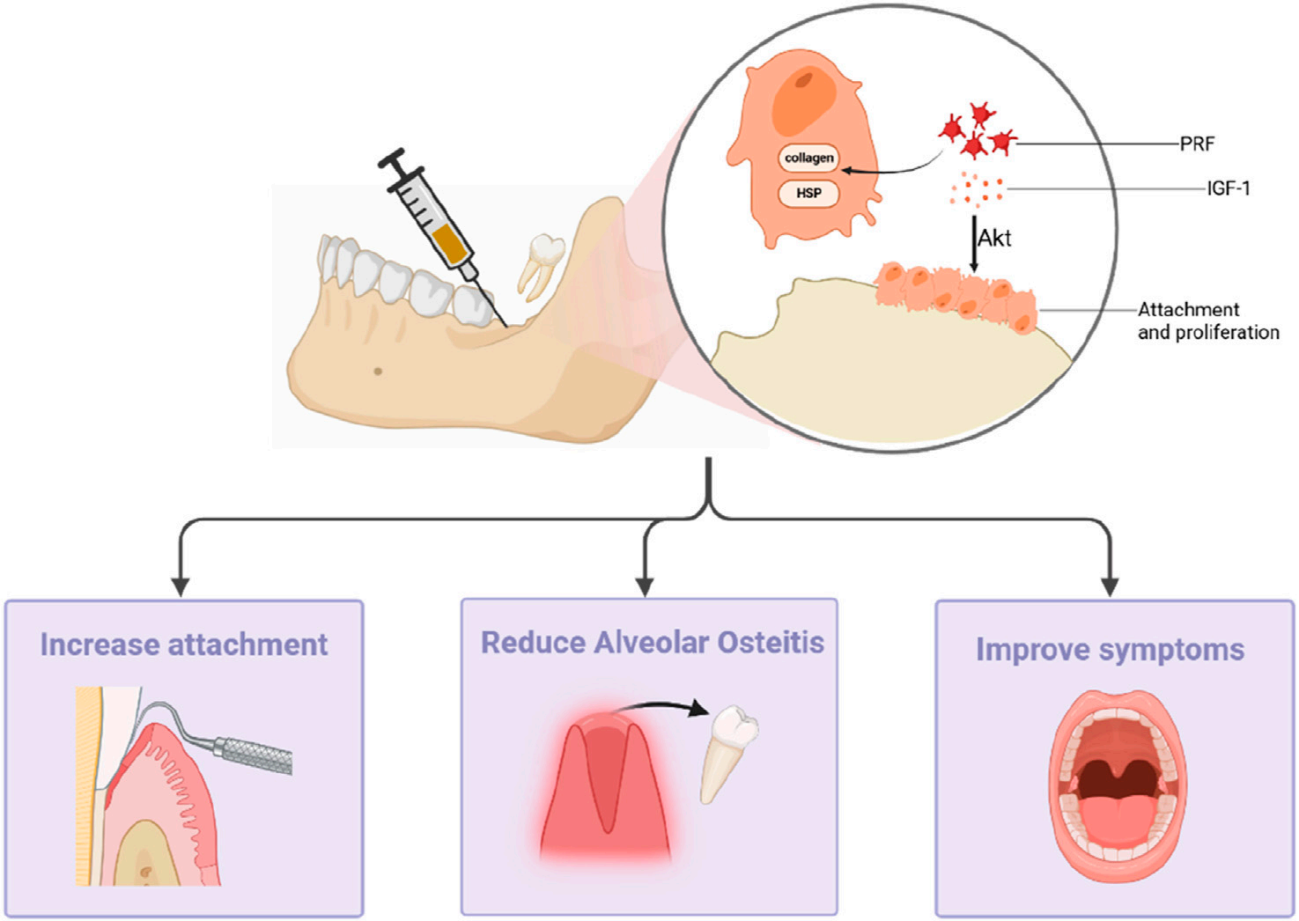
PRF can promote bone healing and regeneration by stimulating osteoblast attachment and proliferation via the Akt pathway and by activating heat shock proteins and collagen synthesis proteins. It can increase gum attachment, reduce the risk of alveolitis, and reduce postoperative complications.

The extraction of the third molar is a commonly performed procedure in the field of oral surgery. Nevertheless, the planning of this surgical intervention is essential, taking into consideration both the indications for extraction and the inherent procedural risks. Tooth extraction inevitably leads to the absorption of alveolar bone and soft tissue, consequently instigating the remodeling process within the alveolar bone structure. This process significantly impacts the patient’s recovery and subsequent dental interventions. PRF can stimulate increased osteoblast attachment and proliferation through the Akt pathway. Furthermore, PRF promotes bone healing and regeneration by activating heat shock and collagen synthesis proteins, which, in turn, facilitate the synthesis of the bone matrix (Zhang et al., 2012) (Figure 3). In clinical trials, Canellas et al. (2020) reported significant benefits associated with L-PRF in alveolar preservation, reducing horizontal absorption below the alveolar crest. Furthermore, 3 months later, L-PRF can inhibit the vertical absorption of the vestibular wall and increase the total bone volume. Additionally, their histomorphometric analysis revealed a higher percentage of new bone formation when L-PRF was used as a socket filling material after tooth extraction. In a study by Gasparro et al. (2020) on the clinical effects of L-PRF treatment on the distal periodontal pocket of the mandibular second molar after the extraction of impacted third molars, the application of PRF showed statistically significant clinical attachment gains and a reduction in the probing depth.

### Reducing complications after tooth extraction

Following tooth extraction surgery, the activation of the fibrinolytic pathway can occur due to jawbone trauma and bacterial presence. This activation can lead to the dissolution of a blood clot, ultimately resulting in the development of alveolar osteitis (AO), which is a condition characterized by pain and disruptions in daily life. Recently, research has shown that the use of PRF during tooth extraction surgery can effectively reduce the incidence of AO (Asif et al., 2023). Moreover, it can reduce the use of antibiotics, thereby minimizing the systemic side effects associated with their use (Donmezer and Bilginaylar, 2021). PRF can also facilitate periodontal soft tissue recovery and reduce certain postoperative complications. The experimental results from Afat et al. (2019) indicate that L-PRF, either alone or in combination with hyaluronic acid (HA), effectively promotes mucosal healing, preventing mandibular third molar+0 postoperative alveolar osteitis and infections. Recent systematic reviews on PRF indicate that it is most effective during the early healing period, from around 2–3 months after tooth extraction (Al-Maawi et al., 2021). The use of PRF after the extraction of mandibular third molars has been found to improve patients’ pain, swelling, and limited mouth opening capacity; reduce the occurrence of a dry socket; and promote soft tissue healing (Bao et al., 2021). However, the concentration of PRF can influence the natural healing process and, thus, affect treatment outcomes. Through quantitative experiments, Jasmine et al. (2022) found that low-dose PRF effectively enhances the natural healing cascade, while high doses interfere with the natural healing process.

### Producing better results combined with other materials

L-PRF has been employed in alveolar socket preservation following tooth extraction. While the efficacy of PRF as a standalone treatment is not significantly superior to the other commonly used grafting materials, combining PRF with other materials demonstrates greater therapeutic advantages compared to using a single material alone. Some studies have compared the therapeutic outcomes of PRF with the other commonly used grafting materials after tooth extraction and have also investigated the combined effects of PRF with other medications. In contrast to more commonly employed grafting materials like xenograft (Elbrashy et al., 2022) and concentrated growth factor (Li et al., 2022), PRF is somewhat less effective. A systematic review has demonstrated that PRF, whether used independently or in combination with other grafting materials, yields favorable outcomes in socket preservation surgery (Alrayyes and Al-Jasser, 2022). When compared to cases where no grafting is performed, the application of PRF has exhibited statistically significant results. Combining PRF with graft materials yields better results compared to using them separately.

## Dental implant surgery

**FIGURE 4:**
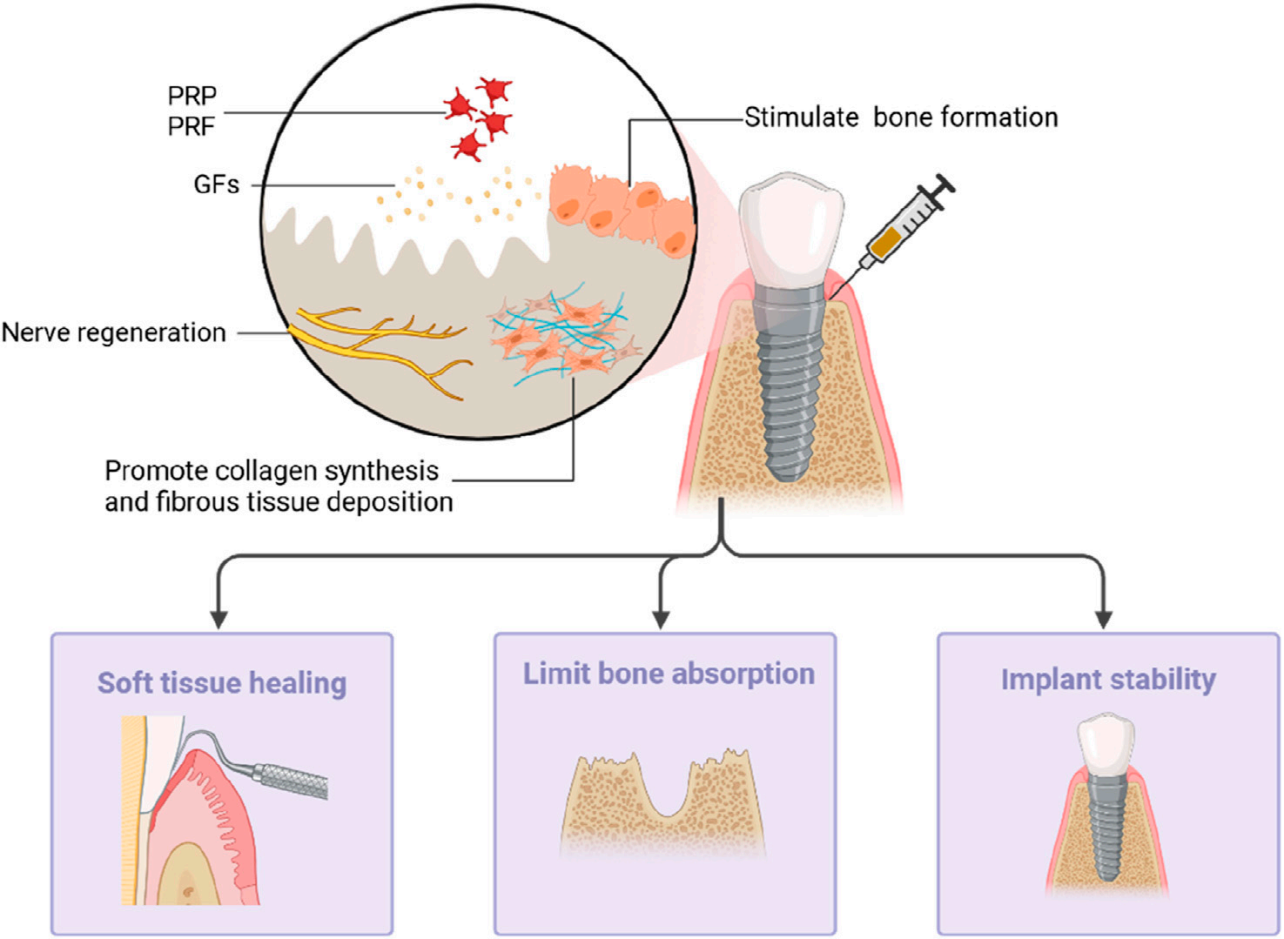
PRP and PRF can stimulate collagen fiber synthesis and fiber tissue deposition at the implant site by releasing growth factors to promote bone formation and nerve regeneration. It can promote the healing of the surrounding soft tissues, limit bone resorption, and maintain implant stability.

Dental implant surgery is a common procedure conducted by oral and maxillofacial surgeons. Dental implants support fixed prostheses or removable dentures in cases of missing teeth. The key determinant of a successful implant is osseointegration, denoting the intimate contact between the bone and the implant. However, it is common to encounter an alveolar ridge with a reduced height or width at the site of a missing tooth, which can significantly impact the osseointegration process. Several studies have explored the application of PCs to guide and enhance bone formation, proving to be a viable strategy for improving both the quality and volume of bone at the implant site (Walch et al., 2024; Qu et al., 2021) (Figure 4).

### Promote bone repair and healing of surrounding tissue

PRP releases a large amount of growth factor that promotes collagen synthesis and stimulates the deposition of fibrous tissue and bone formation at the implantation site, thereby aiding bone repair. Huang et al. (2019)’s preclinical study using micro-computed tomography on beagle dogs showcased the positive impact of PRP on the volume and structure of peri-implant trabecular bone, further supporting the potential of PRP to enhance bone and adjacent tissue recovery. PRP contains growth factors that can enhance the survival of nerve cells, thus promoting nerve regeneration. Animal experiments conducted by Song et al. (2019) showed the significant effect of PRP on the diameter of myelinated nerve fibers, providing potential for PRP in repairing nerve damage caused by implant surgery.

The effect of PRF on the healing of bone and soft tissue around the implant is more obvious. PRF gradually releases growth factors and can act for longer. Kargarpour et al. (2020) and colleagues found that PRF membranes effectively inhibit the formation of osteoclasts from hematopoietic progenitors in bone marrow cultures, indicating its inhibitory effect on osteoclastogenesis *in vitro*. Notably, PRF consistently and more strongly promoted the proliferation and differentiation of rat calvarial osteoblasts than PRP did (He et al., 2009). Sharma et al. (2023) compared the changes in soft and hard tissues around dental implants implanted with PRF. The experimental results indicated that PRF promotes healing in both bone and the surrounding soft tissues. The study found that the experimental group using PRF had less alveolar bone loss and minimal changes in the soft tissues, with significant differences observed in the probing depth and papilla index scores regarding soft tissue changes. X-ray evaluations showed that the experimental group had smaller crestal bone changes compared to those of the control group. Both groups exhibited statistically significant differences in crestal bone changes in the X-ray assessments. In addition, Al-Diasty et al. (2022) observed that a highly embedded PRF membrane can increase the width of keratinized mucosa around the implant, offering advantages such as reduced operation time and less postoperative discomfort and pain.

### Enhance the stability of the implant

PRF combined with other medications can more effectively limit bone absorption around implants. The application of Sticky Bone/PRF around implants inhibits bone changes compared to using PRF alone, promoting long-term implant success (El Shafei et al., 2022). PRF can also enhance early implant stability, which is closely associated with the quality of bone formation. An *in vivo* study by Anapu et al. (2024) demonstrated that coating implants with PRF on the surface can improve the osseointegration capability of the implants. In sinus augmentation procedures, PRF can also enhance the quantity and quality of bone formation, thereby improving implant stability (Guan et al., 2023; Chitsazi et al., 2018). However, this does not increase the survival rate of implants (Guan et al., 2023; Sivakumar et al., 2023).

## Temporomandibular joint disorder

Temporomandibular joint disorder (TMD) is a pathological condition affecting the temporomandibular joint, chewing muscles, and associated structures. The prevalence of TMD in the population is 30%–40% (Schiffman et al., 2014). The onset of TMD is hidden at the early stage, and in its advanced stage, it can cause severe damage to the structure and function of the temporomandibular joint, gradually resulting in functional impairments related to eating, speech, and breathing and ultimately leading to significant dental and facial deformities, thereby markedly diminishing an individual’s quality of life.

### Combined with arthrocentesis

Arthrocentesis is a minimally invasive, simple, and effective TMD treatment method, which typically involves inserting a cannula into the upper joint space between two different puncture sites. It can inhibit the inflammation mediators and cytokines from causing pain, as well as minimize friction between the joint surfaces. PRP have demonstrated excellent therapeutic effects on TMD, alleviating patients’ pain, improving the maximum degree of mouth opening, reducing the sounds made by the joint, and enhancing their chewing efficiency. Sikora et al.'s clinical pathological series study (Sikora et al., 2022) indicated that five sessions of PRP treatment exhibited a more pronounced immediate analgesic effect (71% joint improvement) than the improvement in the maximum degree of mouth opening (53% patient improvement). In Ansar et al. (2022)’s study, the comparison between intra-articular PRP injections and saline injections revealed PRP’s significant advantages in pain relief and joint sound improvement. Rajput et al. (2022)’s study revealed that the former had a higher success rate in pain relief, while PRP demonstrated superior effectiveness in addressing joint noise and mandibular deviation. When used in conjunction with arthrocentesis, PRP and PRF can have a better effect on promoting movement and alleviating pain. Some studies combined joint puncture and PRP injections and found that the combination showed the best results in terms of pain symptoms (Abbadi et al., 2022; Işık et al., 2023). Several studies have compared the therapeutic efficacy of PRP with other medications, finding that an intra-articular PRP injection is more effective than intra-articular betamethasone, sodium hyaluronate, and local anesthesia combined with hydrocortisone (Prakash et al., 2022; Gupta et al., 2018).

### Combined with HA

**FIGURE 5:**
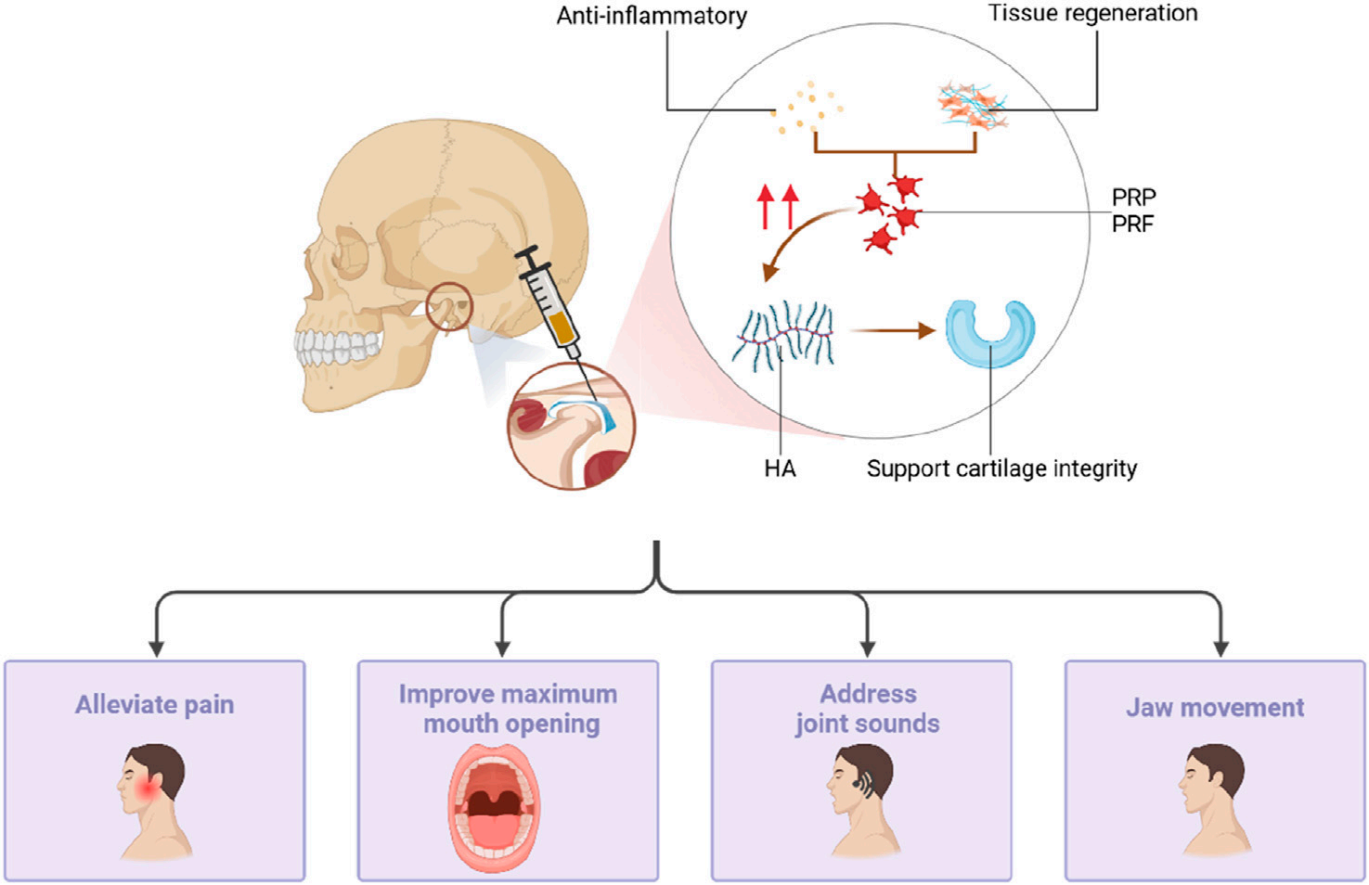
PRP can also be injected into the temporomandibular joint by joint puncture. PRP has accelerated tissue regeneration and anti-inflammatory properties, which can enhance the protective effect of HA on cartilage by promoting chondrocyte viability and collagen synthesis, reducing pain, improving mouth opening, addressing joint sounds, and jaw movement.

PRP not only demonstrates good therapeutic efficacy when used alone but also shows significant advantages when combined with other medications (Figure 5). HA can support cartilage integrity and prevent degenerative joint diseases such as osteoarthritis. PRP accelerates tissue regeneration and possesses anti-inflammatory properties (Hegab et al., 2023). It enhances the protective effect of HA on cartilage by promoting chondrocyte vitality and collagen synthesis. Toameh et al. (2019) compared the therapeutic effects of an intra-articular PRP injection and HA, revealing a significant increase in the maximum degree of mouth opening in both the PRP and HA groups compared to that of the control group. Moreover, the PRP group exhibited significantly better results in pain intensity and chewing efficiency compared to the HA and control groups. Simultaneously, the injection of PRP + HA after joint puncture significantly improved the maximum degree of voluntary mouth opening, pain index scores, and joint sounds (P < 0.005) (Harba and Harfoush, 2021; Hegab et al., 2023), ameliorating the symptoms in patients with TMD.

### PRF shows long-term efficacy

Moreover, PRF has been shown to provide superior long-term efficacy compared to PRP. Xu et al. (2023)’s systematic review and network meta-analysis suggested that PRP and PRF exhibit similar short-term efficacy in treating TMD pain and the maximum degree of mouth opening, with PRF showing long-term efficacy. Additionally, compared to PRP, injecting PRF is more effective in treating joint clicking sounds in patients with internal temporomandibular joint disease (Manafikhi et al., 2022).

### Limitations of PRP

However, PRP has drawbacks. Firstly, PRP may induce mild discomfort, swelling at the injection site, and potential adverse reactions, including inflammation. Secondly, PRP necessitates an invasive preparation procedure for patients and is harder to acquire than HA. When devising a treatment plan, the invasiveness of the preparation procedure and the patient’s preferences should be considered. Discrepancies in the PRP preparation [centrifugation protocols, (Ferreira et al., 2023) etc.] and application methods [concentration, injection time, dose (Hahn et al., 2020), etc.] across studies may contribute to the varying experimental outcomes. Therefore, the establishment of a standardized protocol for PRP preparation and application is imperative.

## Periodontal tissue regeneration

**FIGURE 6:**
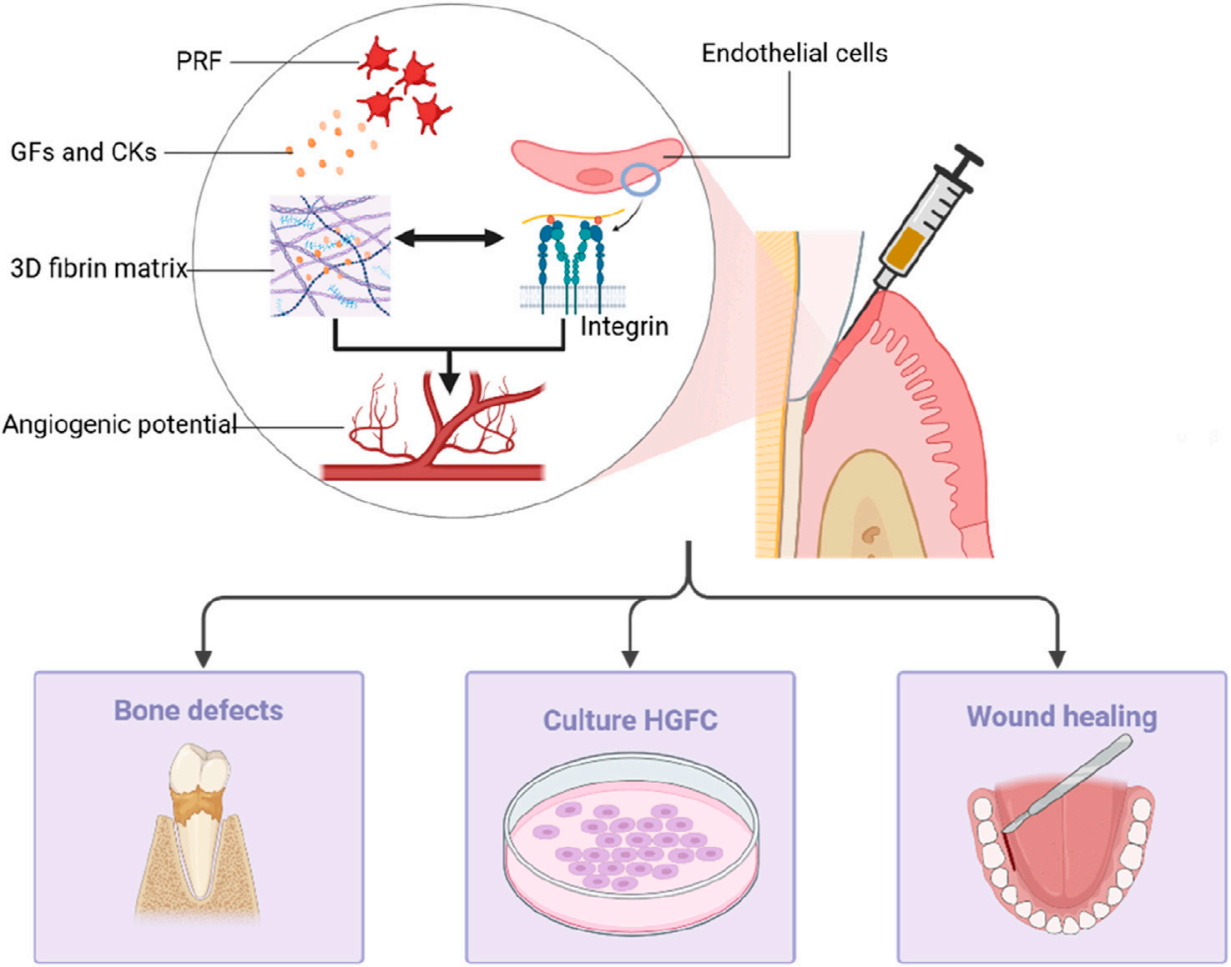
The angiogenesis potential of PRF is the result of endothelial cell interaction with 3D fibrin matrix through endothelial cell expression of integrins, which can facilitate applications in the treatment of bone defects, *in vitro* culture of HGFC, and in promoting postoperative healing of gum surgery.

PRP and PRF are becoming more prominent in regenerative medicine (Miron et al., 2016). These platelet concentrates enhance soft tissue healing and cause periodontal tissue regeneration through their tissue-inducing properties. PRF exhibits the capability to diminish pro-inflammatory cytokines, promote angiogenesis, eliminate bacteria, and improve clinical outcomes for bone formation and filling (Figure 6).

### In intraosseous defects and class II bifurcation defects

Periodontal regeneration techniques are applied to intrabony and Class II furcation defects to promote tissue regeneration. PRF has emerged as a valuable regenerative biomaterial in this field. Specifically, in the treatment of mandibular molar Class II furcation defects, the addition of an autogenous bone graft to PRF can significantly promote clinical attachment and reduce the probing depths (Serroni et al., 2022). Furthermore, in intrabony defect treatment, PRF combined with open flap debridement (OFD) demonstrates more clinical advantages over OFD alone by reducing defect depth and supporting bone regeneration (Padrón-Molina et al., 2023; Baghele et al., 2019). However, these studies have high heterogeneity, emphasizing the need for improved randomization and blinding protocols for new clinical trials to obtain better conclusions.

### In the promotion of gum tissue regeneration

PRF provides autologous growth factors that enhance cell migration, proliferation, and soft tissue regeneration. Its regenerative properties are attributed to its angiogenic potential, largely facilitated by a 3D fibrin matrix. This matrix not only plays a key role in clot formation but also acts as a carrier for cytokines and growth factors like VEGF, IGF, TGF-β1, and PDGF, which are crucial for tissue repair. During angiogenesis, endothelial cells express αvβ3 integrin, enabling interaction with fibrin, fibronectin, and vitronectin within the matrix. Furthermore, *in vitro* studies reveal that L-PRF significantly accelerates the culturing of human gingival fibroblasts (HGFCs), with HGFC proliferation shown to increase proportionally with L-PRF concentration (Mudalal et al., 2021). These findings highlight the concentration-dependent regenerative potential of PRF, warranting further exploration in clinical settings.

Injectable PRF can modify the gingival phenotype, enhancing the gingival thickness and preventing recession (Manasa et al., 2023). İzol and Üner (2019) conducted free gingival grafting surgery on patients with gingival recession and found that the application of injectable PRF on the root surface may have a positive impact on the closure of the root surface. Subsequently, Albatal et al. (2023) discovered further advancements in reducing the recession depth, increasing the width of the attached gingiva, and accelerating wound healing by adding liquid PRF to the root surface in conjunction with free gingival grafting. Studies have also revealed that the application of PRF membrane in patients undergoing free gingival grafting surgery can improve postoperative wound healing in the palate region and enhance patients’ quality of life. A systematic review by Meza-Mauricio et al. (2021) suggested that the collective evidence supports the use of PRF membrane after free gingival graft harvesting in the palate region to facilitate wound healing, reduce postoperative discomfort pain, and eliminate postoperative bleeding. However, this study has limitations, including a small number of randomized clinical trials and the high heterogeneity among PRF protocols, indicating the need for further in-depth research.

## Conclusion

Overall, PCs in the oral and maxillofacial regions exhibit promising multifunctionality in the treatment of tissue lesions. PCs promote bone healing, markedly enhance patients’ recovery from medication-related osteonecrosis, accelerate healing following tooth extraction trauma, and aid in implant surgery. They also play a vital role in addressing temporomandibular joint disorders and periodontal tissues. Regarding therapeutic efficacy, PCs demonstrate significant benefits both as a standalone treatment and in synergy with other therapeutic modalities. However, there are still some limitations to PCs at present. The preparation of platelet concentrates is unstandardized, and the heterogeneity in various experiments is considerable. Factors such as the preparation process, the components of PCs, and whether the platelets are activated significantly influence their therapeutic effectiveness. The clinical efficacy and feasibility of PC formulations may be inconsistent. It is imperative to acknowledge that PCs could serve as a disease-modifying drug, acting to counteract important aspects of osteoarthritis pathophysiology (cartilage breakdown, inflammation, and bone remodeling). However, their efficacy in slowing the progression of osteoarthritis remains unproven (Simental-Mendía et al., 2023; Hegab et al., 2015). Future research and practice should deepen our understanding of PCs, refine the preparation and treatment methods for platelet concentrates, conduct meticulously designed randomized trials with increased sample sizes, enhance experimental research reliability, and establish a robust foundation for their extensive application in the medical field.

## Abbreviations

PCs, Platelet concentrates; PRP, Platelet-rich plasma; PRF, Platelet-rich fibrin; GFs, Growth factors; MRONJ, Medication-related osteonecrosis of the jaw; AO, Alveolar osteitis; HA, Hyaluronic acid; TMD, Temporomandibular joint disorder; OFD, Open flap debridement; HGFCs, Human gingival fibroblasts.
